# Olive Oil: Processing Characterization, and Health Benefits

**DOI:** 10.3390/foods9111612

**Published:** 2020-11-06

**Authors:** Dimitrios Boskou, Maria Lisa Clodoveo

**Affiliations:** 1 School of Chemistry, Aristotle University of Thessaloniki, 54124 Thessaloniki, Greece; 2 Interdisciplinary Department of Medicine, University of Bari, 70121 Bari, Italy

**Keywords:** extra virgin olive oil, varietal authentication, Nuclear Magnetic Resonance spectroscopy, pigments, polar lipids, ultrasound, bio-phenols, pharmaceutical molecular mechanisms of action, primary public health prevention strategies, healthy aging and longevity

## Abstract

The Mediterranean diet is now well known worldwide and recognized as a nutrition reference model by the World Health Organization. Virgin olive oil, prepared from healthy and intact fruits of the olive tree only by mechanical means, is a basic ingredient, a real pillar of this diet. Its positive role in health has now been a topic of universal concern. The virtues of natural olive oil, and especially of extra virgin olive oil, are related to the quality of the fruits, the employment of advanced technologies, and the availability of sophisticated analytical techniques that are used to control the origin of the fruits and guarantee the grade of the final product. With the aim of enriching the recent multidisciplinary scientific information that orbits around this healthy lipid source, a new special issue of *Foods* journal has been published.

This Special Issue collected specific articles from different areas of research. To produce a high-quality oil it is necessary to start from the right selection of cultivars in the olive production region. Therefore, the Special Issue dedicates an article to the varietal authentication of extra virgin olive oils [1]. The proposed approach combines the quantification of the total and positional fatty acids in the triacylglycerols fraction, as well as volatile compounds. The analysis is combined with chemometric methods in order to classify Italian extra virgin olive oil (EVOO) monocultivar samples. The developed method indicates a higher discriminant power that can be used to contrast frauds. To complete the picture of analytical tools, a second article discusses the application of the Nuclear Magnetic Resonance (NMR) technique. NMR spectroscopy coupled with multivariate analysis is a powerful analytical tool to check geographic characterization of commercial extra virgin olive oil [2].

Virgin olive oil is a phytocomplex, a product containing hundreds of non-glyceridic substances that are responsible for the coloring of the product or contribute to the hedonistic value. They may also have antioxidant or other properties beneficial to health. An important class of such compounds is the pigments. The methods for determining them were the subject of a specific article that describes how to quantify the total amount of carotenoids and chlorophyll derivatives [3].

The wide range of molecules present in olives and virgin olive oil also includes polar lipids such as glycerophospholipids and glycolipids. A specific article describes findings on the identification and characterization of such polar lipids, their potentiality as markers of the identity and traceability of olive oil, and their potential impact on nutrition and health [4].

The best oils of all time are the oils produced in the last 20 years thanks to the improvement of hygiene standards and the development of technological innovations. The application of ultrasound to olive paste is among the most relevant innovations. It started from research laboratories and reached the market. The strength of this innovative process lies in the change of traditional milling. The device that combines ultrasound with heat exchange significantly increases the quantity of oil produced and the level of biologically important phenols [5].

Studies of the favorable effects of olive oil bioactive ingredients are continuously opening new paths for medical and pharmaceutical research. A special chapter of this Issue discusses pharma-nutritional evidence that indicates how EVOO bio-phenols might exert important physiological actions that bring about cardioprotection, chemoprevention, and a lower incidence of neurodegeneration. The study focuses on recent findings that elucidate their molecular mechanisms of action [6].

The consumption of dietary fats, which occur naturally in various foods, poses an important impact on health. The last contributions reports the results of the adults from Athens metropolitan area (ATTICA) (*n* = 1128) and the MEDiterranean Islands Study (MEDIS) (*n* = 2221 adults from various Greek islands) studies. The use of olive oil in food preparation and the bio-clinical characteristics of the Greek participants were investigated in relation to successful aging. It is suggested that primary public health prevention strategies to promote healthy aging and longevity should encourage the enhanced adoption of practices based on the exclusive use of olive oil for culinary purposes [7].
